# Supplementary material for: How do we classify organ involvement in Chagas disease? A systematic review of organ involvement since 1909, Highlighting the urgent need for a universal classification system in Chronic Chagas disease
Source: PLoS Negl Trop Dis. 2024 Aug 5;18(8):e0012367. doi: 10.1371/journal.pntd.0012367 (PMC11326633; doi:10.1371/journal.pntd.0012367)
Supplement: S1 Table — (DOCX) [file pntd.0012367.s001.docx]

How do we classify organ involvement in Chagas disease?

Supplementary Table 1. Search engine

| Database | Search terms | Number of articles | after eliminating duplicates |
| --- | --- | --- | --- |
| [PubMed](https://drive.google.com/file/d/1--yiDcGj0dLhQB85XwoxoCbe25eSqdJs/view?usp=sharing) | (("Chagas Disease"[MeSH Terms] OR "chaga*"[Title/Abstract] OR "Trypanosomiasis"[MeSH Terms] OR "trypanosom*"[Title/Abstract]) AND ("Classification"[Title/Abstract] OR "Classifications"[Title/Abstract]) AND "humans"[MeSH Terms]) AND (humans[Filter]) | 174 | 116 |
| [Cochrane_rev](https://drive.google.com/file/d/1eQpzD0wz1A7lyd2mdhM0_LMfhb8Oe-Bz/view?usp=sharing) | “Chagas’ disease” in All Text AND “Chagas disease” in All Text AND “Chagas” in All Text AND “American trypanosomiasis” in All Text - (Word variations have been searched) | 3 | 3 |
| [Cochrane_trials](https://drive.google.com/file/d/1yL0rgKYzKyY5IZnKbQ9ArY1qXirOWtAz/view?usp=sharing) |  | 6 | 6 |
| [Scopus](https://drive.google.com/file/d/1sQAS5Y_ZFRfPff-yO-ITlsSIygMOyKaI/view?usp=sharing) | (TITLE-ABS-KEY(chagas disease) OR TITLE-ABS-KEY(american trypanosomiasis)) AND (TITLE-ABS-KEY(classification) OR TITLE-ABS-KEY(classifications)) AND ( LIMIT-TO ( SUBJAREA,"MEDI" ) ) | 714 | 613 |
| [Embase](https://drive.google.com/file/d/1TOcbfM30mMSXECNtE-U58baiT9tjFCCc/view?usp=sharing) | 'chagas disease':ti,ab,kw AND 'classification':ti<,ab,kw  AND 'human'/de | 127 | 123 |
| [LILACS](https://drive.google.com/file/d/1wUQd_IHKoyky8jpJECT-6T76tLSB85A4/view?usp=sharing) | (chaga$ or trypanosomiasis) AND (classification$) | 400 | 349 |
| [Web of science](https://drive.google.com/file/d/1XYv6xyk2d-pXIk2PH_q4Zh15GtU2MwUP/view?usp=sharing) | TS=(( ("Chagas Disease" OR chaga* OR Trypanosomiasis OR trypanosom*) AND (Classification OR Classifications) )) | 545 | 437 |
| Total |  | 1969 |  |
| duplicates |  |  | 750 |
| Discarded by title |  | 1058 |  |
| Sought for retrieval |  |  | 161 |
|  | Reports not retrieved | 20 |  |
| Assessed for eligibility |  |  | 141 |
| Added from bibliography |  |  | 25 |
| TOTAL [Reviewed References](https://docs.google.com/spreadsheets/d/13lWCaeWYZ16GGgTiCPHtacnOaYLmYOVOWHjT7YH_pY4/edit#gid=0) |  |  | 166 |
|  | Excluded for not including CD classification | 73 |  |
|  | Excluded for not including original CD Classification | 66 |  |
|  | Included as relevant to the research question |  | 27 |
