# Supplementary material for: How do we classify organ involvement in Chagas disease? A systematic review of organ involvement since 1909, Highlighting the urgent need for a universal classification system in Chronic Chagas disease
Source: PLoS Negl Trop Dis. 2024 Aug 5;18(8):e0012367. doi: 10.1371/journal.pntd.0012367 (PMC11326633; doi:10.1371/journal.pntd.0012367)
Supplement: S2 Table — (DOCX) [file pntd.0012367.s002.docx]

**Supplementary Table 2.Chagas Classifications**

| Year (ref) | Title | Cardiologic | Digestive | Other | Symptoms | ECG | Holter | Chest X-ray | LVC* | Myocardial biopsy | Echocardiogram | Natriuretic peptides | MRI | Esophagogram | Barium enema | Esophageal manometry | Endoscopy |
| --- | --- | --- | --- | --- | --- | --- | --- | --- | --- | --- | --- | --- | --- | --- | --- | --- | --- |
| 1916(1) | Pathogenic processes of American trypanosomiasis | **✔** | **x** | **✔** * ** | **✔** | **x** | **x** | **x** | **x** | **x** | **x** | **x** | **x** | **x** | **x** | **x** | **x** |
| 1960(2) | Clinical and radiological aspects of aperistalsis of the oesophagus | **x** | **✔** | **x** | **x** | **x** | **x** | **x** | **x** | **x** | **x** | **x** | **x** | **✔** | **x** | **x** | **x** |
| 1966 | Clinical and epidemiological study of chronic heart involvement in Chagas (3) | **✔** | **x** | **x** | **✔** | **✔** | **x** | **x** | **x** | **x** | **x** | **x** | **x** | **x** | **x** | **x** | **x** |
| 1967(4)& 1968(5) | The various types of chagasic myocarditis 1 & 2 | **✔** | **x** | **x** | **✔** | **✔** | **x** | **✔** | **x** | **x** | **x** | **x** | **x** | **x** | **x** | **x** | **x** |
| 1974(6) | Clinical aspects of Chagas Disease | **✔** | **✔** | **✔** * | **x** | **x** | **x** | **x** | **x** | **x** | **x** | **x** | **x** | **x** | **x** | **x** | **x** |
| 1979(7) | A review of the anatomo-clinical classification of Chagas' disease | **✔** | **✔** | **✔** * | **✔** | **✔** | **x** | **✔** | **x** | **✔** | **x** | **x** | **x** | **x** | **x** | **x** | **x** |
| 1982(8) | Electrocardiographic classification and abbreviated lead system for population-based studies of Chagas' disease | **✔** | **x** | **x** | **x** | **✔** | **x** | **x** | **x** | **x** | **x** | **x** | **x** | **x** | **x** | **x** | **x** |
| 1985(9) | Evaluation of Cardiac Function by Radioisotopic Angiography, in Patients with Chronic Chagas Cardiopathy | **✔** | **x** | **x** | **✔** | **✔** | **x** | **✔** | **x** | **x** | **x** | **x** | **x** | **x** | **x** | **x** | **x** |
| 1982(10) | Left ventricular cineangiography in Chagas' disease: Detection of early myocardial damage | **✔** | **x** | **x** | **✔** | **✔** | **x** | **x** | **✔** | **x** | **x** | **x** | **x** | **x** | **x** | **x** | **x** |
| 1985(11) | Life expectancy analysis in patients with Chagas' disease: prognosis after one decade (1973–1983) | **✔** | **x** | **x** | **✔** | **✔** | **x** | **x** | **✔** | **x** | **x** | **x** | **x** | **x** | **x** | **x** | **x** |
| 1987(12) | Clinical, histochemical, and ultrastructural correlation in septal endomyocardial biopsies from chronic chagasic patients: Detection of early myocardial damage | **✔** | **x** | **x** | **✔** | **✔** | **x** | **x** | **✔** | **✔** | **x** | **x** | **x** | **x** | **x** | **x** | **x** |
| 1995(13) | Clinical Classification of Chronic chagasic Cardiomyopathy. XXII Congress of Cardiology International Symposium on Chagas Disease | **✔** | **x** | **x** | **✔** | **✔** | **x** | **✔** | **x** | **x** | **x** | **x** | **x** | **x** | **x** | **x** | **x** |
| 1996(14) | Report of the 1995 World Health Organization/International Society and Federation of Cardiology Task Force on the Definition and Classification of cardiomyopathies | **✔** | **x** | **x** | **x** | **x** | **x** | **x** | **x** | **x** | **x** | **x** | **x** | **x** | **x** | **x** | **x** |
| 2003(15) | Proposed classification of chagasic megacolon by opaque enema | **x** | **✔** | **x** | **x** | **x** | **x** | **x** | **x** | **x** | **x** | **x** | **x** | **x** | **✔** | **x** | **x** |
| 2005(16) | Application of the new classification of heart failure (ACC/AHA) in chronic chagasic heart disease: critical analysis of survival curves. | **✔** | **x** | **x** | **✔** | **✔** | **x** | **x** | **x** | **x** | **✔** | **x** | **x** | **x** | **x** | **x** | **x** |
| 2005(17) | Brazilian Consensus on Chagas Disease | **✔** | **✔** | **x** | **✔** | **✔** | **x** | **x** | **x** | **x** | **✔** | **x** | **x** | **✔** | **x** | **x** | **x** |
| 2006(18) | Clinical forms of *Trypanosoma cruzi* infected individuals in the chronic phase of Chagas disease in Puebla, Mexico | **✔** | **✔** | **x** | **✔** | **✔** | **x** | **✔** | **x** | **x** | **✔** | **x** | **x** | **✔** | **✔** | **✔** | **x** |
| 2007(19) | Assessment of Myocardial Damage in Chronic chagasic Patients using QRS Slopes | **✔** | **x** | **x** | **✔** | **✔** | **✔** | **x** | **x** | **x** | **✔** | **x** | **x** | **x** | **x** | **x** | **x** |
| 2008(20) | Prognostic value of natriuretic peptides in Chagas' disease: a 3-year follow-up investigation | **✔** | **x** | **x** | **✔** | **✔** | **x** | **x** | **x** | **x** | **✔** | **✔** | **x** | **x** | **x** | **x** | **x** |
| 2010(21) | Chagas Disease | **✔** | **x** | **x** | **✔** | **✔** | **✔** | **x** | **x** | **x** | **✔** | **x** | **x** | **x** | **x** | **x** | **x** |
| 2011(22) | I Latin American guidelines for the diagnosis and treatment of chagas' heart disease | **✔** | **x** | **x** | **✔** | **✔** | **x** | **✔** | **x** | **x** | **✔** | **x** | **x** | **x** | **x** | **x** | **x** |
| 2012(23) | Endoscopic classifications of esophageal changes in chagasic megaesophagus | **x** | **✔** | **x** | **x** | **x** | **x** | **x** | **x** | **x** | **x** | **x** | **x** | **x** | **x** | **x** | **✔** |
| 2016(24) | 2 nd Brazilian Consensus on Chagas Disease | **✔** | **✔** | **x** | **✔** | **✔** | **x** | **x** | **x** | **x** | **✔** | **x** | **x** | **✔** | **x** | **x** | **x** |
| 2021(25) | New Classification for Esophageal Motility Disorders (Chicago Classification Version 4.0) | **x** | **✔** | **x** | **x** | **x** | **x** | **x** | **x** | **x** | **x** | **x** | **x** | **x** | **x** | **✔** | **x** |
| 2023 (26) | SBC Guideline on the Diagnosis and Treatment of Patients with Cardiomyopathy of Chagas  Disease – 2023 | **✔** | **x** | **x** | **✔** | **✔** | **✔** | **✔** | **x** | **x** | **✔** | **x** | **✔** | **x** | **x** | **x** | **x** |

LVC: left ventricular cineangiography; MRI: magnetic resonance imaging; * Neurologic; ** Suprarrenal; ✔ indicates that the classification includes information displayed in the respective column. **x** indicates that the classification DOES NOT include information displayed in the respective column.

**Bibliography**

1. Chagas C. Processos patojenicos da tripanozomiase americana. Memórias do Instituto Oswaldo Cruz. 1916.

2. de RJ, Lauar KM, de OA. [Clinical and radiological aspects of aperistalsis of the esophagus]. Rev Bras Gastroenterol. 1960 Sep-Dec;12:247-62.

3. JJ P, JR R, HG B, JA S, CG Y. Clinical and epidemiological study of chronic heart involvement in Chagas'. D - 7507052. 1966 (- 0042-9686 (Print)):T - ppublish.

4. L MJ. Los diversos tipos de miocarditis chagásica 1. Prensa Méd Arg 54. 1967.

5. L MJ. Los diversos tipos de miocarditis chagásica 2. Prensa Méd Arg 54. 1968.

6. Aspectos clínicos de la enfermedad de chagas. Pan American Health Organization, 1974.

7. Curti HJ, Sanches PC, Bittencourt LA, Carvalhal Sdos S. [A review of the anatomo-clinical classification of Chagas' disease]. Arq Bras Cardiol. 1979 Oct;33(4):277-81.

8. Maguire JH, Mott KE, Souza JA, Almeida EC, Ramos NB, Guimarães AC. Electrocardiographic classification and abbreviated lead system for population-based studies of Chagas' disease. Bull Pan Am Health Organ. 1982;16(1):47-58.

9. Kuschnir E, Sgammini H, Castro R, Evequoz C, Ledesma R, Brunetto J. “[Evaluation of Cardiac Function by Radioisotopic Angiography, in Patients with Chronic Chagas Cardiopathy].”. Arquivos brasileiros de cardiologia. 1985.

10. Hugo A.G. Carrasco JSB, George Inglessis, Abdel Fuenmayor, César Molina,. Left ventricular cineangiography in Chagas' disease: Detection of early myocardial damage. American Heart Journal. 1982;104(3):595-602.

11. Espinosa R, Carrasco HA, Belandria F, Fuenmayor AM, Molina C, González R, et al. Life expectancy analysis in patients with Chagas' disease: prognosis after one decade (1973–1983). International Journal of Cardiology. 1985 1985/05/01/;8(1):45-56.

12. Carrasco Guerra HA, Palacios-Prü E, Dagert de Scorza C, Molina C, Inglessis V G, Mendoza RV. Clinical, histochemical, and ultrastructural correlation in septal endomyocardial biopsies from chronic chagasic patients: Detection of early myocardial damage. American Heart Journal. 1987;113(3):716-24.

13. Infecciosas CAdCyC. Clasificación Clínica de la Miocardiopatía Chagásica Crónica. XXII Congreso de Cardiología Simposio Internacional de Chagas. 1995.

14. Richardson P, McKenna W, Bristow M, Maisch B, Mautner B, O'Connell J, et al. Report of the 1995 World Health Organization/International Society and Federation of Cardiology Task Force on the Definition and Classification of cardiomyopathies. Circulation. 1996 Mar 1;93(5):841-2.

15. Silva ALd, Giacomin RT, Quirino VdA, Miranda ESd. Proposta de classificação do megacólon chagásico através de enema opaco. Rev Col Bras Cir. 2003 2003/02;30(1):4-10.

16. Xavier SS, Sousa ASd, Moreno AH. Aplicação da nova classificação da insuficiência cardíaca (ACC/AHA) na cardiopatia chagásica crônica: análise crítica das curvas de sobrevida. Rev SOCERJ. 2005 2005/06;18(3):227-32.

17. Ministério SdVeSd. Consenso brasileiro em Doenca de Chagas Revista da Sociedade Brasileira de Medicina Tropical. 2005;38.

18. Sánchez-Guillén MDC, López-Colombo A, Ordóñez-Toquero G, Gomez-Albino I, Ramos-Jimenez J, Torres-Rasgado E, et al. Clinical forms of Trypanosoma cruzi infected individuals in the chronic phase of Chagas disease in Puebla, Mexico. Mem Inst Oswaldo Cruz. 2006 2006/11;101(7):733-40.

19. Pueyo E, Laciar E, Anzuola E, Laguna P, Jane R. Assessment of Myocardial Damage in Chronic Chagasic Patients using QRS Slopes. Computers in Cardiology 2007, Vol 34. 2007;34:725-+.

20. Moreira Mda C, Heringer-Walther S, Wessel N, Moreira Ventura T, Wang Y, Schultheiss HP, et al. Prognostic value of natriuretic peptides in Chagas' disease: a 3-year follow-up investigation. Cardiology. 2008;110(4):217-25.

21. Rassi A, Jr., Rassi A, Marin-Neto JA. Chagas disease. Lancet. 2010 Apr 17;375(9723):1388-402.

22. de Andrade JP, Neto JAM, de Paola AAV, Vilas-Boas F, Oliveira GMM, Bacal F, et al. I Latin American guidelines for the diagnosis and treatment of chagas' heart disease. Executive summary. Arquivos Brasileiros de Cardiologia. 2011;96(6):434-42.

23. Morita S, Morita FH, de Godoy JMP. Endoscopic classifications of esophageal changes in chagasic megaesophagus. Central European Journal of Medicine. 2012 Oct;7(5):596-8.

24. Dias JC, Ramos AN, Jr., Gontijo ED, Luquetti A, Shikanai-Yasuda MA, Coura JR, et al. 2 nd Brazilian Consensus on Chagas Disease, 2015. Rev Soc Bras Med Trop. 2016 Dec;49Suppl 1(Suppl 1):3-60.

25. Herbella FAM, Malafaia O, Patti MG. New Classification for Esophageal Motility Disorders (Chicago Classification Version 4.0(C)) and Chagas Disease Esophagopathy (Achalasia). Abcd-Arquivos Brasileiros De Cirurgia Digestiva-Brazilian Archives of Digestive Surgery. 2021;34(4).

26. Marin-Neto JA, Rassi A, Jr., Oliveira GMM, Correia LCL, Ramos Júnior AN, Luquetti AO, et al. SBC Guideline on the Diagnosis and Treatment of Patients with Cardiomyopathy of Chagas Disease - 2023. Arq Bras Cardiol. 2023 Jun 26;120(6):e20230269.
