# Supplementary material for: How do we classify organ involvement in Chagas disease? A systematic review of organ involvement since 1909, Highlighting the urgent need for a universal classification system in Chronic Chagas disease
Source: PLoS Negl Trop Dis. 2024 Aug 5;18(8):e0012367. doi: 10.1371/journal.pntd.0012367 (PMC11326633; doi:10.1371/journal.pntd.0012367)
Supplement: Attachment — Submitted filename: Response PLOS PNTD-D-23-01525.pdf [file pntd.0012367.s003.pdf]

Dear Dr. Dutra,

Thank you for your recent communication regarding the manuscript titled *"How do we classify organ involvement in Chagas disease? A Systematic Review of Organ Involvement Since 1909, Highlighting the Urgent Need for a Universal Classification System in Chronic Chagas Disease"* submitted for consideration at PLOS Neglected Tropical Diseases.

We are grateful for the diligent review process conducted by the editorial board and independent reviewers. Their feedback is invaluable in enhancing the quality and relevance of our manuscript. We are fully committed to addressing all concerns and suggestions raised during the review process to enhance the clarity, comprehensiveness, and scientific rigor of our work.

Please note that detailed responses to the reviewers' comments can be found below this letter. While we deeply appreciate the contributions of the reviewers and acknowledge their expertise in the field, we wish to respectfully express disagreement with certain opinions expressed in the reviews. We are prepared to address these disagreements in a constructive and detailed manner, providing well-founded justifications and scientific evidence to support our position.

Should you require any further information or clarification, please do not hesitate to contact us.

Sincerely,

Irene Losada Galván

Methods

Reviewer #1: Yes, goals clearly and objectively defined, despite not having a specific hypothesis being tested.
Study design quite appropriate, by using systematic revision, without resource to statistical tools because not warranted.
Articles pertaining to the populations studies not entirely appropriate, as detailed below.
Neither sample size nor statistical analysis are applicable to the subject.
No concerns about regulatory or ethical issues.

Reviewer #2: see comments

Results
-Does the analysis presented match the analysis plan?
-Are the results clearly and completely presented?
-Are the figures (Tables, Images) of sufficient quality for clarity?

Reviewer #1: I have some remarks regarding these aspects. Please see below.

Reviewer #2: see comments

Conclusions
-Are the conclusions supported by the data presented?
-Are the limitations of analysis clearly described?
-Do the authors discuss how these data can be helpful to advance our understanding of the topic under study?
-Is public health relevance addressed?

Reviewer #1: For the conclusions topic I also have some points to be considered by the Authors. Please see belox.

Reviewer #2: see comments

Editorial and Data Presentation Modifications?

Use this section for editorial suggestions as well as relatively minor modifications of existing data that would enhance clarity. If the only modifications needed are minor and/or editorial, you may wish to recommend “Minor Revision” or “Accept”.

Reviewer #1: I would recommend minor revision by the authors, regarding a few points:
1 - In the Introduction section, it is stated that “Predicting the individual probability of disease progression remains a challenge, as there are currently no reliable tools available for this purpose”. It would be relevant to emphasize that although there is no clinical or laboratory predictor of how the disease will evolve in a specific individual, the

mean probability of progression from the indeterminate form to the cardiomyopathy form has been recently defined by a systematic review and subsequent metanalysis. JAMA Netw Open. 2020 Aug 3;3(8):e2015072. Moreover, the mortality risk in patients already running through the cardiomyopathy form was also assessed using similar tools. Chadalawada et al. ESC Heart Fail. 2021 Dec;8(6):5466-5481.

Thank you for your comment. The point of this paragraph is precisely the lack of tools for individual risk prediction. The first proposed reference, although interesting, reports on the pooled risk of progression to a cardiac form over time in people in the indeterminate or acute phase. However, this study does not resolve the lack of tools for individual probability of progression.
Similarly, we do not consider it relevant to include the proposed reference on mortality prediction, as our study focuses on elements of progression within the disease, rather than on predictors of mortality, for which there is indeed much more literature.

2 - In the same section it is stated that "There are several clinical classifications for the visceral involvement of CD, but four of them stand out, all staging Chagas disease with a focus on heart disease: Modified Los Andes (10), Kuschnir (11), 2nd Brazilian consensus (12) and I Latin American guidelines (13). All these classifications, attempt to stratify patient's prognosis based on different information such as ECG, chest X-ray, echocardiogram, and clinical heart failure symptoms for cardiomyopathy, among others". However, another classification, that of Rassi et al in The Lancet 2010, would merit to be quoted as such, according to the various aspects as analyzed later by the authors, in the text of their manuscript. I would suggest that the authors acknowledge that fact and possibly have a more critical take on the merits and limitations of each classification.

Indeed, on reflection, Rassi's classification deserves to be mentioned with the main ones. It has been modified in the text (line 88, new reference 14).

3 - The authors also state that "Following the classification of chronic heart failure (CHF) published in 2001 by the ACC/AHA(45), from which CD was excluded, a 2005 study(35) aimed to evaluate the prognostic performance of this classification applied to a cohort of patients with CD, Table 8. In a 5-year follow-up, no progression to Chronic Chagas Cardiomyopathy (CCC) was observed among patients who had a normal ECG at baseline. Therefore, only patients with an abnormal ECG at baseline were included. Patients were divided into 4 groups according to the results of ECG, echocardiogram, and clinical symptoms of heart failure. Survival curves showed a large difference between groups B and C, so group B was subdivided according to LVEF measured by echocardiography; this was the first classification to include a non-invasive measurement of ventricular capacity in Chagas." This is not correct, Kuschnir used RNA to measure EF non-invasively, the method only requires an intravenous injection of the radionuclide agent, not intracardiac contrast ventriculography. In fact, the RNA should be considered the gold standard for measurement of both right and left ventricular ejection fraction, because it is quite more accurate than the echocardiographic technique. I also suggest the replacement of the word "capacity" by "systolic function" in the context.

"An invasive procedure is one where purposeful/deliberate access to the body is gained via an incision, percutaneous puncture, where instrumentation is used in addition to the puncture needle, or instrumentation via a natural orifice" as per BMJ Open. 2019; 9(7): e028576.
We can therefore agree that a method that "only" requires intravenous injection of a

radionuclide agent is an invasive method. Looking again at the methods in Kuschnir's original paper, he describes the performance of radiocardiography and radioisotope angiography, both of which require intravenous administration of radioisotopes. We agree that RNA should probably be considered the gold standard for measuring ventricular ejection fraction. However, the distinction between this method and echocardiographic measurement is beyond the scope of this paper.

The word "capacity" was replaced by "systolic function" in line 315.

3 - On what basis the review paper by Bern C et al published in 2007 by JAMA was excluded from the present review, by Losada et al? In that paper, following a systematic review of evidence, a detailed diagnostic basis for detecting the absence or presence of heart and digestive organ involvement is presented and also is used for prognostic and therapeutic measures in people with Chagas disease.

Although the authors consider the C Bern systematic review to be fundamental, it is not included in this review, as only the original classifications of Chagas disease are included, as stated in line 137.

4 - Another striking oversight by Losada et al efers to the AHA and InterAmerican Society of Cardiology statement published in 2018 in Circulation by Pereira Nunes et al, summarizing the most updated information on diagnosis and screening of patients after T, cruzi infection, focusing primarily on its cardiovascular aspects. Is the oversight justifiable?

Similarly, the article referred to has not been included because it is not an original classification.

Incidentally, and even if we appreciate all the comments from the reviewer, we do not consider constructive the use of use of the word "oversight" in the context of revising a manuscript.

5 - A recent e-book edited by two of the coauthors of the systematic review (MJPD and JG) - https://doi.org/10.1007/978-3-030-44054-1- included what is probably the most accurate and updated take on the esophageal and colon complications caused by Chagas disease. In the chapter entitled “Chagas Disease: An Unknown and Neglected Disease” results of studies for characterization of esophageal disorders in T. cruzi-infected individuals were reported in endemic and non-endemic countries, as well as the prevalence and severity of colonic involvement by de Oliveira RB et al. Could the authors of the manuscript reconsider and add that relevant information content?

The book mentioned above is, again, an excellent overview, but it does not contain any original classifications.

Reviewer #2: see comments

Summary and General Comments

Use this section to provide overall comments, discuss strengths/weaknesses of the study, novelty, significance, general execution and scholarship. You may also include additional comments for the author, including concerns about dual publication, research ethics, or publication ethics. If requesting major revision, please articulate the new experiments that are needed.

Reviewer #1: It is unfortunate that the recently published Guideline of the Brazilian Society of Cardiology on the Diagnosis and Treatment of Patients with Cardiomyopathy of Chagas Disease – Arq Bras Cardiol 2023 has not been included in the systematic methodology review carried out by the authors of the manuscript. Notwithstanding the circumstance that the whole guideline publication appeared only in June 2023, the Authors stated in their Abstract that "We searched relevant electronic medical databases from their inception dates to July 2023". Also, an executive summary of the Guideline had already been published as a preprint in the Arquivos Brasileiros de Cardiologia, by December 2022. In addition, one of the coauthors of the manuscript was an active collaborator for the elaboration of the Guideline, rendering this oversight even more surprising and regretful.

Although the overall paper, for the purposes of the authors, stands well without that last guideline, I would suggest that they could reconsider, and give the manuscript the chance of being enriched by the addition of the data conveyed by this last guideline.

We have carefully considered your comments and have now included the recently published Guideline of the Brazilian Society of Cardiology on the Diagnosis and Treatment of Patients with Cardiomyopathy of Chagas Disease in our review. Although it is based on an existing classification, it incorporates new elements such as magnetic resonance results (new reference 43, included in Supplementary 2 -former table 1-,figure 2, table 15 and lines 418-424)

In addition to that general suggestion, I have some few relevant remarks to be considered by the authors, should they agree to perform a major revision:
In essence, the authors documented the lack of generally accepted criteria for the diagnosis and prognosis of organ involvement in Chagas disease, especially in regard to the cardiomyopathy form. They call for a standardization of criteria that does not exist in the realm of the various classification and other related papers they systematically reviewed. I am afraid that when published, as it deserves to be, this paper will sound rather anodyne and missing the opportunity to perform a more critical review of the criteria they identified in the classifications analyzed. I know that the review aimed solely at reporting on the diverse criteria used in various classifications of organic involvement due to Chagas disease. That goal was indeed accomplished. However, without a more critical appraisal of the data, the highly categorized authors of the investigation may have missed a good opportunity of a real advance in the field. Ideally, their team could have come up with some proposition that would reflect a synthesis of the heterogenous data assembled and perhaps become more widely accepted.
Nevertheless, provided that Losada and collaborators include in their revision the recent guideline of the Brazilian Society of Cardiology just published, it is quite possible that it could constitute a starting point for the proposal here suggested. In fact the new Brazilian guideline compiled data from a more extensive array of documents than that surveyed by Losada et al. A thorough review of diagnostic methods for detection of structural and functional cardiac abnormalities was presented, with a heart failure classification dedicated to CD peculiarities added. Detection of rhythm derangements and

thromboembolic complications was also fully addressed. Also, the guideline extensively dwelled on a critical appraisal of risk stratification methods for prognosis of patients with Chagas disease.

While we appreciate your perspective, we respectfully disagree with the notion that our work missed an opportunity for a more critical review. The primary objective of our systematic review was to document the existing criteria for classification of organ involvement in Chagas disease. We aimed to provide a comprehensive overview of the diverse criteria utilized across various classifications, which we believe is a crucial first step in addressing the lack of standardization in this field.

It is essential to recognize that synthesizing heterogeneous data and proposing universally accepted criteria require a robust foundation of evidence, which our review sought to establish. However, we agree that the next step in advancing the field involves evaluating real patient data to determine which classification or elements thereof are most clinically relevant. We are committed to pursuing further research in this direction and appreciate your insights into potential avenues for future investigation.

Reviewer #2: The MS intitled "How do we classify organ involvement in Chagas disease? A Systematic Review of Organ Involvement Since 1909, Highlighting the Urgent Need for a Universal Classification System in Chronic Chagas Disease" by Losada I et al., submitted to PLoSNTD as a Research Article, cannot be accepted in the present format and content, based on the following considerations:

1- The title confuses and misleads readers. As Chagas disease is a complex, systemic disease, that may have outcome influenced by non-genetic factors of host (as nutritional, pre-immunological status) and genetic factors of host and parasite, is there a need for a universal classification of Chagas disease? If so, it is not this "Systematic Review" that highlights it, but rather the current needs of patients, the barriers faced by professionals in the healthcare system and the people who spend effort in clinical trials.

We respectfully disagree with the assessment that the manuscript's title is confusing or misleading. The title accurately reflects the focus of our systematic review, which is to examine the classification of organ involvement in Chagas disease and underscore the urgent need for a universal classification system. While we acknowledge the complexity of Chagas disease and the multitude of factors that can influence its outcome, our review specifically addresses the lack of consensus in classifying organ involvement, which can impede clinical decision-making and hinder research efforts.

2- The classification of the MS as a "Research Article" is not correct, as it is a review on a subject or could be a "Political Platform". In fact, this last format may call attention to the need for an international effort focused on Chagas disease as a systemic disorder with different clinical forms. Also, considering the contribution of aspects of the host (genetic, nutritional condition) and the infecting parasite population to clinical outcomes, there are aspects to be completely identified before classification. I agree that Chagas disease deserves greater attention to clinical outcomes and clinical classification (cardiac, digestive, cardi-digestive, neurological and even behavioral, as indicated more recently; for example, see Silva et al., 2020, focused on depression) to allow proper diagnosis and care. Thus, a more political perspective may add call more attention to this point.

While we appreciate the alternative perspective suggested, we respectfully disagree with the assertion that our manuscript should be classified as a "Political Platform" rather than a research article. Our work represents a systematic review that includes a critical assessment of the uptake of each classification, providing valuable insights into the current landscape of organ involvement classification in Chagas disease. Furthermore, we acknowledge the importance of considering various factors, including genetic and nutritional conditions of the host, as well as characteristics of the infecting parasite population, in understanding clinical outcomes. However, we believe that our focus on synthesizing existing knowledge and proposing the need for a universal classification system aligns with the objectives of a research article. We also welcome the suggestion that the article be accompanied by a letter to the editor highlighting the importance of the issue, which we would be happy to write if the editorial team deems it appropriate.

3- Carlos Chagas paid attention to several clinical aspects of the acute phase of the disease he described: the common symptoms of acute infection; a systemic disease (the mistake with goiter, has been already overcome, as a consensus); the cardiac acute form; and the acute nervous form (particularly, in children). All these are crucial contributions of Chagas, 1916. But the authors, more fixed to timeline, jumped to an article describing the cardiac form in the chronic phase (not even the first one to describe the heart disease). The clinical points of the acute phase, crucial for diagnosis and care, were not dissected. It is a point to be revised.

Although it is mentioned several times in the text, it may not have been made sufficiently clear that the focus of this work is on the chronic phase. Therefore, the acute phase work mentioned above was not considered relevant. Therefore, the word “chronic” has been added in line 107 of the Methods section.

4- Considering the “proposed aim” “organ involvement”, the neurological form (neurobehavioral form), mostly as a sequelae of stroke, is often the first clinical sign of the chronic Chagas disease, crucial for diagnosis in people under 40’, as it is a crucial point to be considered in Latin America (and, particularly Latin American individuals residing in North America, Europe and Oceania). This point has not been properly discussed.

Although the authors agree that stroke may be one of the first manifestations of the disease, it is not so clear that this manifestation is an entity of the infection itself, that is, due to the direct action of *T. cruzi* on the central or peripheral nervous system, but a consequence of the prothrombotic state of the disease or a result from the thromboembolic event. On the other hand, the presence of the parasite is necessary for the cardiac or digestive tissue damage characteristic of Chagas' disease to occur. However, there is manifestation in the form of chagomas in the central nervous system, in the context of 'reactivation' in immunocompromised patients, which is outside the scope of this study.

It is important to remember that this study is limited to collecting and contextualizing the existing classifications of chronic involvement, which do not include a neurological form, probably for the reasons given above.

5- The “timeline format” should be summarized. Although interesting, it is not the

crucial point of this MS (for example, line 149 to 270). The authors should focus on the consensus (or lack of consensus) of the last two decades.

While we appreciate the input, we respectfully disagree with the proposal to condense the timeline format. This format offers crucial historical insight, illustrating the evolution of consensus (or lack thereof) regarding organ involvement classification in Chagas disease. Notably, some existing classifications, especially those pertaining to digestive involvement, have been in use for over two decades. We firmly believe that understanding the historical progression of classifications is essential for gaining a comprehensive perspective on the subject.

6- The format of this revision, the selected words, the presentation of the MS is a pitfall, considering the so good group of researchers involved in this task and the relevance of the theme. The selected words (Chagas disease, Trypanosoma cruzi, classification) may have brought a huge number of articles on Trypanosoma cruzi classification (DTUs, for example), and excluded another group of articles on clinical classification, clinical forms, the supposed target of interest. Indeed, it seems to be the case as, for example, seminal articles on classification of Chagas disease cardiopathy and cardiac form of Chagas disease by Laranja et al, and Viotti et al., are not part of this "review". It brings the main problem: the selection of words for a "Systematic review". Anyone that confronts the problem of clinical classification of Chagas disease knows the importance of the work published by Laranja et al., Circulation, 1956, studying almost 1000 patients (120 acute form, others chronic) to the recognition of the acute and, particularly, the chronic cardiac form of Chagas disease. So, the exclusion of this article (available online) is a failure and argues that the selection of words was not adequate. Or, more likely, the format of the review. A narrative review based on the experience of this qualified team and focused on the relevant objective would be more appropriate and valid for current Chagas disease research and advocacy needs.

While we recognise that papers such as Laranja's are fundamental to the understanding of the disease, it does not meet the selection criteria as it does not present an original classification.

Indeed, the search engine returned inapplicable results (as with any systematic review), but it is precisely the experience of the qualified team that produced this paper that can ensure that no exclusion of relevant classifications, past or present, is discernible.

7- This MS did not include the recent (June 2023, the cutoff was July 2023) article by Marin-Neto JA et al., Arq Bras Cardiol. 2023 Jun 26;120(6):e20230269. doi: 10.36660/abc.20230269.

We appreciate the reviewer's suggestion and have included this work in the text: new reference 43.

8- In fact, the relevance of the topic "standardization of clinical classification" was not properly explored, considering the (recognized) need for basis to support the advocacy of public health policies, allowing affected patients access to the appropriate diagnosis of the clinical form, appropriate treatment and supports for decisions in clinical trials. The presentation of the MS (abstract, author summary) misguides the reader to something that is not delivered.

We respectfully disagree with the assertion that the relevance of the topic "standardization of clinical classification" was not adequately explored in our manuscript. Our aim was to provide a comprehensive review of existing classifications of organ involvement in Chagas disease and highlight the urgent need for a universal classification system.

9- The initial question (How do we classify organ involvement is Chagas disease?) assumes that attention will be given to “organ involvement”, however it is not the case. Imaging approaches (and exams) and biomarkers are not adequately discussed. Furthermore, hematological and immunological involvement, generally the patients' first access in a public health service, are not considered.

This assertion is completely outside the scope of this review. If biomarkers or immunological changes have not been addressed in this review, it is because none of the current or previous clinical classifications are based on these principles. The authors recognise the immense importance of these elements (which some of us are actively investigating). However, organ involvement in Chagas disease usually refers to visceral cardiac and gastrointestinal changes.

10- The MS should be rewritten as a “narrative review”, a “point of view” or a “policy platform”, including the tables that summarize the data originally published in Spanish or Portuguese (a relevant point of the MS). Although a historical point of view is important, the MS lacks a more recent view of key issues and a clear presentation of current gaps, lack of consensus, and proposal of procedures needed to fill gaps in knowledge.

The authors consider that the methodology used leaves no doubt that this is a systematic review. However, as discussed in an earlier response, we are prepared to include a letter to the editor highlighting the implications of the lack of a generally accepted classification.

The tables presented already include the summary of some of the papers originally published in Spanish or Portuguese.

While we understand the value of providing a current perspective on key issues, we believe that work such as this is the first step in thinking about this issue and drawing conclusions. However, we believe that gaps in knowledge and regulatory gaps in clinical trials or in regulatory documents are clearly identified.

11- A crucial point, before considering prognosis, one should consider the progression of Chagas disease, since the ratio of progression of IND to CARD is 1 to 8%/year, depending on the group of patients studied (parasite/host genetics, host nutritional condition, previous immunological conditions). More, these crucial points regarding the different classifications of the Brazilian Consensus (2005) and the First Latin American guidelines (2011) were not considered.

Although we recognize the importance that classifications have in relation to prognosis, which is why they group patients with similar prognoses together, we do not see the relevance of delving deeper into this issue in our work.

In relation to the classifications proposed in the "Brazilian Consensus on Chagas disease (2005), ratified in 2016, and the "I Latin American Guidelines", ratified in 2023, we provide a brief explanation of the difference in the staging of groups "A'," B1" and "B2", proposed by both. Please see lines 388-402.

12- Prognosis is a very broad topic and deserves a discussion in the “Limitations” section, since good reviews have focused on this subject and a consensus awaits reliable biomarkers.

The assessment of prognosis and biomarkers is outside the scope of this paper.

13- As a first approach to the topic (very relevant), and considering the qualified team of authors, and particularly the last part of the discussion session, I consider that the modified version of the MS should be submitted as a “platform policy”, trying to pave the way for an international effort to establish a consensus on the clinical classification of Chagas disease, based on anamnesis (including clear obligatory questions), non-invasive and accessible tests and clear interpretations of the results (considering that a well acquired and analyzed EKG may contribute enormously to diagnosis and prognosis). It will be a shame to miss the opportunity to propose a “comprehensive review” by presenting this pile of tables that will not attract readers.

This reviewer hopes that, although this initial step may be hard, the authors present a revised version of the MS that may contribute to better and accessible monitoring and management of Chagas disease patients in non-endemic and, particularly, endemic areas

We agree, as we have said before, that this issue needs accompanying policy efforts. We would be happy to write further papers in this regard. However, if no effort is made to collect and analyse data (sometimes even a "pile of tables" is necessary), no scientifically convincing arguments can be constructed.

However, in order to facilitate reading and to lighten the content of the manuscript, the authors have decided to move the old Table 1 to the Supplementary Material 2 and to replace it with the new Figure 2, which summarizes the parameters included in the different classifications that make up this work.

Minor comments:

1- Chagas disease has two clinical phases: acute and chronic. The “indeterminate phase” (line 518) has been excluded, by consensus.

We have made the necessary change from "Indeterminate phase" to "Indeterminate form" throughout the manuscript as per your recommendation (new line 541)

2- Line 155: “sudden;” (what?) sudden was the death of this sentence.

“death” was added at the end of the sentence (line 165)

3- Patients suffering from a cardiac or digestive form of Chagas disease may be asymptomatic, therefore “asymptomatic” (line 163) is not a condition, but a transient state and clinical examinations may reveal signs of a clinical form.

In around 60% of patients infected with *T.cruzi*, the indeterminate (and asymptomatic) state is not transient but their definitive state. However, for the sake of clarity, since the meaning of “indeterminate” for Dr. Chagas was different to the current definition: "asymptomatic" has been replaced by "disease-free" (line 173)

4- Most tables could be moved to Supplementary Materials. Tables 21 and 22 with number of citations and arithmetic mean (last columns) are not relevant. The number of citations is a metric used for academic purposes. The relevance of a Guideline is assessed by its use, generally adopted by professionals and health care systems, to standardize procedures.

We agree that there is no standard measure of uptake of classifications. Therefore, the text specifies that these are only proxies to quantify the degree of uptake. Table 1 is now supplementary material 2.

5- LVEF (left ventricular ejection fraction), is defined only in line 540, but used several times before.

The acronym has been developed in the first appearance in the text (line 313).

6- Myocarditis – written twice (line 152)

repetition has been removed (line 162)
